# Sodium-Glucose Co-transporter Type-2 Inhibitors’ Effect on Quality of Life in Older Adult Population

**DOI:** 10.7759/cureus.47005

**Published:** 2023-10-13

**Authors:** Duygu Tutan, Ayşe Erdoğan Kaya, Mehmet Kayaalp

**Affiliations:** 1 Department of Internal Medicine, Erol Olçok Training and Research Hospital, Çorum, TUR; 2 Department of Psychiatry, Erol Olçok Training and Research Hospital, Çorum, TUR

**Keywords:** older adults, geriatry, quality of life, sodium-glucose cotransporter, diabetes

## Abstract

Introduction

The global elderly population is expanding, with chronic conditions like diabetes diminishing their quality of life. Sodium-glucose co-transporter type 2 (SGLT-2) inhibitors hold promise in improving quality of life by addressing hypervolemia, obesity, and lipid irregularities. However, these drugs can lead to adverse effects, such as polyuria, dehydration, and weight loss, which may detrimentally impact older patients. We aimed to investigate the association between SGLT-2 inhibitors and quality of life in older adults with diabetes.

Methods

The research included 100 type II diabetes mellitus patients over 65, without active infections, malignancies, immunodeficiencies, and hematological disorders. Fifty patients were using empagliflozin or dapagliflozin and 50 patients were using other oral antidiabetics for at least six months. Patient demographics, laboratory studies, drug usage and side effects, additional diseases, Geriatric Depression Scale scores, and World Health Organization Quality of Life OLD (WHOQoL-OLD) module scores were noted.

Results

No significant difference between gender distribution, SGLT usage, chronic disease existence, chronic disease count, depression scores, or incidents of chronic diseases other than hyperlipidemia was observed. Hyperlipidemia incidence was significantly higher in the SGLT group, while other laboratory parameters were not statistically significantly different between groups. There were no significant differences in autonomy, past-present-future activities, social skills, death, intimacy, and total WHOQoL-OLD scores between the two groups. However, there were statistically significantly worse outcomes in patients with at least one SGLT adverse effect in terms of sensory quality of life scores. Dehydration existence was negatively correlated with lower autonomy, PPF activities, and total quality of life scores. Multivariate linear regression analysis showed no significant differences in the total WHOQoL-OLD score after adjusting for confounding factors.

Conclusion

Age and depression remained the main factors affecting the quality of life in diabetic patients. SGLT-2 inhibitor side effects did not decrease the quality of life in older individuals, who are more prone to unfavorable consequences.

## Introduction

The older adult population is increasing globally with the prolongation of life expectancy. Chronic diseases, especially diabetes, constitute an important global health problem in older adults [1,2]. Diabetes significantly affects the quality of life, which is defined as the individual's perception of himself in relation to his goals, interests, and expectations in the culture in which he lives, and causes an increase in morbidity and mortality [3,4].

In older individuals, quality of life gradually decreases over the years due to both the physical problems of old age and the burden of diabetes and diabetes-related complications [4]. Moreover, the presence of diabetes in older adults has been associated with deteriorated quality of life, independent of other chronic conditions and geriatric syndromes [5]. The development of new treatment strategies that will improve the quality of life in this population is very important for people to reduce fragility and to have a healthy aging process, as well as to reduce global costs and health burdens [6].

In addition to providing glycemic control, sodium-glucose co-transporter type-2 (SGLT-2) inhibitors are known to improve the quality of life in various populations due to their benefits such as reducing hypervolemia, reducing obesity with weight loss, regulating lipid levels by increasing high-density lipoprotein (HDL) levels and decreasing triglyceride levels, and reducing hypertension (HT) [7]. Previous studies in older adult patients have demonstrated cardiorenal protective effects and beneficial effects on cognitive function through antioxidant, antiatherogenic, and direct neuroprotective effects [8,9]. However, in addition to these effects, it has been reported that it may cause undesirable effects such as polyuria, dehydration, increased frequency of genitourinary infections, and weight loss [10]. Geriatric patients may be more sensitive to these effects [11]. Due to these adverse effects associated with SGLT-2 inhibitors, a detailed evaluation is recommended before starting treatment [12]. The literature review demonstrated that there were no studies explicitly addressing the relationship between the effects of SGLT-2 inhibitors and geriatric quality of life. This study aimed to investigate the relationship between SGLT-2 inhibitors and quality of life in the older adult population.

## Materials and methods

This study was planned as a prospective study and approved by the Hitit University Faculty of Medicine Ethics Committee (Decision No: 2023-49, Date: April 11, 2023). All patients who were seen at the Erol Olçok Training and Research Hospital, Department of Medicine outpatient clinic with diabetes between April 2023 and June 2023, were evaluated. Patients over 65 years of age who have been using empagliflozin or dapagliflozin for the last six months without any acute or chronic active infections, active malignancy, immunodeficiency, known hematological disease, known liver failure, who are not receiving renal replacement therapy, who do not have a diagnosis of dementia, pregnancy, or breastfeeding status were included, and patients with type I diabetes mellitus, patients who are using drugs that can exacerbate proteinuria or change lipid levels/proteinuria (statins, ACE inhibitors, SGLT-2 inhibitors, etc.), patients using drugs that alters hemogram parameters (steroids, cytotoxic agents, etc.) and those whose blood results could not be obtained were excluded. During the patient selection process, 26 patients were excluded because they were taking medications that could alter lipid profiles and hemogram parameters. A total of 100 diabetes mellitus type II patients were included in the study, 50 of those patients were using an SGLT-2 inhibitor, and 50 were using other diabetic drugs. The gender, age, diabetes duration, chronic diseases such as HT, coronary artery disease (CAD), congestive heart failure (CHF), chronic kidney disease (CKD), asthma, hypothyroidism, hyperlipidemia (HPL), benign prostatic hyperplasia (BPH), cerebrovascular incident (CVI), and chronic obstructive pulmonary disease (COPD), SGLT adverse effects such as polyuria, dehydration, weight loss, hypoglycemia, and urinary infection, Geriatric Depression Scale (GDS) Scores (and groups according to GDS), World Health Organization Quality of Life OLD (WHOQoL-OLD) assessment results (sensory, autonomy, past-present-future (PPF) activities, social skills, death, intimacy, and total scores), serum white blood cell count (WBC), neutrophil, lymphocyte, monocyte, platelet counts, RDW, MPV, hemoglobin, fasting glucose, creatinine, blood urea nitrogen (BUN), uric acid, albumin, HDL, low density lipoprotein (LDL), total cholesterol, triglyceride, hemoglobin A1c (HbA1c), alanine aminotransferase (ALT) and aspartate aminotransferase (AST) levels, spot urine protein to spot urine creatinine ratio of 100 patients were assessed and recorded.

Patients’ depression levels were assessed with GDS scores. Quality of life measurements were done using the WHOQoL-OLD questionnaire. WHOQoL-OLD is a tool with 24 total questions with six subsections consisting of sensory capability, autonomy, PPF activities, social skills, death-related fears, and intimacy [13]. While higher points in autonomy, PPF activities, social skills, and intimacy mean a higher quality of life, higher scores in sensory capabilities and death-related fears mean a lower quality of life. Thus, the total WHOQoL-OLD score was calculated as “total score = (autonomy score + PPF activities score + social skills score + intimacy score) - (death-related fears score + sensory capability score)”. Patients were divided into two groups for the first analysis: non-SGLT patients and SGLT-using patients. Then SGLT group was also divided into two groups, patients without side effects and patients with side effects, and these groups of two and three were compared in terms of demographic characteristics, laboratory results, depression, and quality of life indicators. After the comparison of variables between main groups was done, comparisons of WHOQoL-OLD parameters for SGLT groups, adverse effects, adverse effect count, depression groups, and adverse effect types were done. A multivariate linear regression analysis was done to examine the association of variables with the total WHOQoL-OLD score.

All statistical analyses were performed using IBM SPSS Statistics for Windows software (Version 26; IBM Corp., Armonk, NY, USA). Descriptive statistics were reported using numbers and percentages for categorical variables. Numerical variables were reported as mean ± standard deviation for Gaussian distributed variables and median value accompanied by minimum and maximum values in parentheses for non-Gaussian distributed variables. Data distribution was evaluated using the Shapiro-Wilks test. Relationships between variables were investigated with Pearson and Spearman correlation coefficients. The comparison of numerical measurements for two independent research groups was made according to distribution. GDS score, WHOQoL-OLD social skills, and death scores, hemoglobin level, lymphocyte, platelet count, total cholesterol, HDL cholesterol, albumin levels were evaluated with the Mann-Whitney U test for two group analysis, and with the Kruskal-Wallis test for three group analysis. Age, diabetes duration, WHOQoL-OLD sensory, autonomy, PPF activities, intimacy, and total scores, HbA1c levels, serum WBC, RDW, MPV, neutrophil, monocyte counts, glucose, BUN, creatinine, uric acid, albumin, AST, ALT, triglyceride, LDL cholesterol levels, spot urea protein to spot urine creatinine ratio was assessed using the student t-test for two group analysis, and analysis of variance test (ANOVA) was used for three group analysis.

A post-hoc analysis with Bonferroni correction was done after the ANOVA. The Chi-Square test was used to evaluate the statistical significance of categorical variable differences across groups. A multivariate linear regression test was used for the multivariate analysis with gender, age, diabetes duration, SGLT usage, side effect status, chronic disease count, GDS score, hemoglobin levels, polyuria, dehydration, weight loss, hypoglycemia, and urinary infection existence as covariates to determine the association between the variables and the Total WHOQoL-OLD Score after adjusting for confounding factors. For the statistical significance level, p < 0.05 was accepted as meaningful.

## Results

A total of 100 patients who were seen at the outpatient clinic were included in the study. Thirty seven (37%) of the patients were male, and 63 (63%) were female. The median age of all patients was 70 years, the youngest patient was 64 years old, and the oldest patient was 86 years old. The median diabetes duration was 12 years (1-33). Eighty one (81%) of the patients had additional chronic diseases. Thirty-seven patients had only one chronic disease, 28 had two, 13 had three, and only three patients had four or more chronic diseases. Sixty-three (63%) patients had HT, 33 (33%) patients had CAD, seven (7%) patients had hypothyroidism, 12 patients had (12%) HPL, and other chronic diseases can be seen in Table 1. The mean GDS Score was 14.88±4.95, and 58 (58%) of the patients were classified in the certain depression group. The median WHOQoL-OLD sensory score was 11 (6-17), median autonomy score was 11 (6-17), median PPF activities score was 11 (4-17), mean social skills score was 11.26±2.29, mean death score was 10.59±3.07, median intimacy score was 12 (8-18), and the median total score was 22 (3-52). Patients were divided into two groups according to SGLT usage.

**Table 1:** Comparison of variables between non-SGLT patients and SGLT group SGLT: sodium glucose co-transporter inhibitor, HT: hypertension, CAD: coronary artery diseases, CHF: congestive heart failure, CKD: chronic kidney disease, HPL: hyperlipidemia, BPH: benign prostate hyperplasia, CVI: cerebrovascular incidence, COPD: chronic obstructive pulmonary disease, GDS: geriatric depression scale, PPF: past-present-future, HbA1c: hemoglobin A1c, WBC: white blood cell count, RDW: red cell distribution width, MPV: mean platelet volume, BUN: blood urea nitrogen, AST: aspartate transaminase, ALT: alanine transaminase, HDL: high-density lipoprotein, LDL: low-density lipoprotein, Prot/Cre: Protein to creatinine ratio

Variables		All Patients	No SGLT	SGLT	Statistical significance
Gender	Male	37 (37%)	15 (30%)	22 (44%)	0.147
	Female	63 (63%)	35 (70%)	28 (56%)	
Age		70 (64-86)	71 (65-86)	67.5 (64-82)	0.005
Diabetes Duration (year)		12 (1-33)	10 (1-33)	15 (1-30)	0.017
Chronic Disease	No additional disease	19 (19%)	7 (14%)	9 (18%)	0.799
	Additional diseases exist	81 (81%)	43 (86%)	41 (82%)	
Chronic Disease Count	None	19 (19%)	10 (20%)	9 (18%)	0.865
	1	37 (37%)	17 (34%)	20 (40%)	
	2	28 (28%)	16 (32%)	12 (24%)	
	3	13 (13%)	6 (12%)	7 (14%)	
	4	3 (3%)	1 (2%)	2 (4%)	
HT	No	37 (37%)	21 (42%)	16 (32%)	0.300
	Yes	63 (63%)	29 (58%)	34 (68%)	
CAD	No	67 (67%)	35 (70%)	32 (64%)	0.523
	Yes	33 (33%)	15 (30%)	18 (36%)	
CHF	No	94 (94%)	46 (92%)	48 (96%)	0.678
	Yes	6 (6%)	4 (8%)	2 (4%)	
CKD	No	95 (95%)	46 (92%)	49 (98%)	0.362
	Yes	5 (5%)	4 (8%)	1 (2%)	
Asthma	No	97 (97%)	48 (96%)	49 (98%)	0.558
	Yes	3 (3%)	2 (4%)	1 (2%)	
Hypothyroidism	No	93 (93%)	45 (90%)	48 (96%)	0.436
	Yes	7 (7%)	5 (10%)	2 (4%)	
HPL	No	87 (87.88%)	48 (96%)	40 (80%)	0.028
	Yes	12 (12.12%)	2 (4%)	10 (20%)	
BPH	No	94 (94%)	46 (92%)	48 (96%)	0.678
	Yes	6 (6%)	4 (8%)	2 (4%)	
CVI	No	96 (96%)	48 (96%)	48 (96%)	1.000
	Yes	4 (4%)	2 (4%)	2 (4%)	
COPD	No	95 (95%)	46 (92%)	49 (98%)	0.362
	Yes	5 (5%)	4 (8%)	1 (2%)	
SGLT Adverse Effects	No adverse effects			15 (30%)	
	At least one effect			35 (70%)	
Adverse Effect Count	0			15 (30%)	
	1			27 (54%)	
	2			7 (14%)	
	3			1 (2%)	
Poliuria	No			30 (60%)	
	Yes			20 (40%)	
Dehydration	No			43 (86%)	
	Yes			7 (14%)	
Weight Loss	No			39 (78%)	
	Yes			11 (22%)	
Hypoglycemia	No			48 (96%)	
	Yes			2 (4%)	
Urinary Infection	No			46 (92%)	
	Yes			4 (8%)	
GDS Score		14.88±4.95	15.08±5.26	14.68±4.67	0.688
Depression Group	No depression	20 (20%)	12 (24%)	8 (16%)	0.051
	Possible depression	22 (22%)	6 (12%)	16 (32%)	
	Certain depression	58 (58%)	32 (64%)	26 (52%)	
Sensory		11 (6-17)	11.5 (6-17)	11 (7-15)	0.397
Autonomy		11 (6-17)	10 (6-16)	11 (7-17)	0.099
PPF Activities		11 (4-17)	11 (8-17)	11 (4-17)	0.541
Social		11.26±2.29	11.08±2.05	11.44±2.52	0.435
Death		10.59±3.07	10.52±3.07	10.66±3.1	0.821
Intimacy		12 (8-18)	12 (8-16)	12 (8-18)	0.883
Total WHO-QoL OLD		22 (3-52)	22 (6-52)	22.5 (3-52)	0.825
HbA1c		7.6 (5-14.2)	7.55 (5.6-14.2)	7.6 (5-11.7)	0.567
WBC		7.74 (4.48-13.97)	7.51 (4.48-12.24)	7.9 (4.69-13.97)	0.167
Hemoglobin		13.62±1.51	12.93±1.44	14.32±1.24	<0.001
RDW		13.7 (11.8-19.2)	13.75 (12-18.6)	13.7 (11.8-19.2)	0.879
Platelet		265.5±76.25	273.78±86.88	257.22±63.71	0.280
MPV		10.6 (8.9-13.1)	10.65 (8.9-13.1)	10.45 (9.2-13)	0.639
Neutrophil		4.54 (1.83-9.4)	4.48 (2.03-9.07)	4.54 (1.83-9.4)	0.521
Lymphocyte		2.36±0.67	2.18±0.64	2.54±0.64	0.006
Monocyte		0.56 (0.31-1.19)	0.51 (0.31-1.09)	0.58 (0.37-1.19)	0.108
Glucose		153.5 (30-574)	146 (30-477)	159.5 (52-574)	0.574
BUN		15 (8-46)	15.5 (8-46)	15 (9-28)	0.357
Creatinine		0.8 (0.5-1.3)	0.9 (0.5-1.3)	0.7 (0.5-1.3)	0.008
AST		18.5 (10-78)	18 (11-46)	19 (10-78)	0.617
ALT		16 (6-63)	16 (6-50)	16.5 (9-63)	0.194
Total Cholesterol		183±40.01	180.74±41.57	185.26±38.68	0.575
Triglyceride		146.5 (51-499)	144 (53-379)	146.5 (51-499)	0.669
HDL		51.83±10.79	51.6±11.62	52.06±10	0.832
LDL		94.5 (30-188)	89 (30-188)	100.5 (40-171)	0.251
Albumin		42.49±3.3	42.58±3.66	42.4±2.93	0.787
Uric Acid		5.1 (2.1-9)	5.25 (2.1-8.6)	5 (2.2-9)	0.202
Spot Prot/Cre		0.13 (0.05-2.42)	0.14 (0.05-2.42)	0.13 (0.05-1.13)	0.378

Comparison between no-SGLT and SGLT-using groups

In the comparative analysis between the two groups, no significant difference was observed between gender distribution (p = 0.147). The median age of patients using SGLT was 67.5 (64-82) years, which was significantly lower than that of patients not on SGLT (p = 0.005). Even though SGLT-using patients were younger than other patients, they had a longer median diabetes duration (15 (1-30) months vs. 10 (1-33) months, p = 0.017). There were no significant differences between chronic disease existence, chronic disease count, and incidents of chronic diseases other than HPL between groups (Table 1). HPL incidence was found to be statistically significantly higher in the SGLT group (20% vs. 4%, p = 0.028).

Laboratory parameters other than hemoglobin level, lymphocyte count, and creatinine levels between groups were also found not statistically significantly different between groups (Table 1). Higher hemoglobin and lymphocyte values were observed in the SGLT group compared to no-SGLT patients (p < 0.001, and p = 0.006, respectively). No-SGLT group’s median creatinine level was 0.9 mg/dL (0.5-1.3), and SGLT-using patients was 0.7 mg/dL (0.5-1.3); while there was a statistically significant difference observed (p = 0.008), but this was not considered a clinical difference. There were no significant differences observed between depression levels (GDS scores) and quality of life parameters (WHOQoL scores) between SGLT and no SGLT groups (p = 0.688 and p = 0.825, respectively).

Comparison between no-SGLT, no side effects, and SGLT side effect groups

When the patients were analyzed in three groups, no significant difference was observed in terms of gender distribution. A significant difference was observed between the groups in terms of the mean age of the patients (p = 0.007). Post-hoc tests showed that this difference was observed between the group without side effects and the no-SGLT group, and there was no significant difference between the other two groups (G1-G2 p = 0.010, G2-G3 p = 0.461, G1-G3 p = 0.158). There were no differences observed in terms of diabetes duration between the three groups (p = 0.057). There were also no differences observed in terms of chronic diseases other than hyperlipidemia (Table 2). Hyperlipidemia incidence was found to be higher in no side effect and side effect groups than in the SGLT group (p = 0.031).

**Table 2:** Comparison of variables between no-SGLT, no side effect, and SGLT side effect groups SGLT: sodium glucose co-transporter inhibitor, HT: hypertension, CAD: coronary artery diseases, CHF: congestive heart failure, CKD: chronic kidney disease, HPL: hyperlipidemia, BPH: benign prostate hyperplasia, CVI: cerebrovascular incidence, COPD: chronic obstructive pulmonary disease, GDS: geriatric depression scale, PPF: past-present-future, HbA1c: hemoglobin A1c, WBC: white blood cell count, RDW: red cell distribution width, MPV: mean platelet volume, BUN: blood urea nitrogen, AST: aspartate transaminase, ALT: alanine transaminase, HDL: high-density lipoprotein, LDL: low-density lipoprotein, Prot/Cre: Protein to creatinine ratio

Variables		No SGLT	No side effects	Side effect observed	Statistical significance
Gender	Male	15 (30%)	6 (40%)	16 (45.71%)	0.325
	Female	35 (70%)	9 (60%)	19 (54.29%)	
Age		71 (65-86)	67 (64-75)	69 (65-82)	0.007
Diabetes Duration (year)		10 (1-33)	15 (7-30)	15 (1-30)	0.057
Chronic Disease	No additional disease	10 (20%)	3 (20%)	6 (17.14%)	0.941
	Additional diseases exist	40 (80%)	12 (80%)	29 (82.86%)	
Chronic Disease Count	None	10 (20%)	3 (20%)	6 (17.14%)	0.822
	1	17 (34%)	8 (53.33%)	12 (34.29%)	
	2	16 (32%)	2 (13.33%)	10 (28.57%)	
	3	6 (12%)	2 (13.33%)	5 (14.29%)	
	4	1 (2%)	0 (0%)	2 (5.71%)	
HT	No	21 (42%)	8 (53.33%)	8 (22.86%)	0.072
	Yes	29 (58%)	7 (46.67%)	27 (77.14%)	
CAD	No	35 (70%)	11 (73.33%)	21 (60%)	0.535
	Yes	15 (30%)	4 (26.67%)	14 (40%)	
CHF	No	46 (92%)	15 (100%)	33 (94.29%)	0.518
	Yes	4 (8%)	0 (0%)	2 (5.71%)	
CKD	No	46 (92%)	15 (100%)	34 (97.14%)	0.354
	Yes	4 (8%)	0 (0%)	1 (2.86%)	
Asthma	No	48 (96%)	15 (100%)	34 (97.14%)	0.727
	Yes	2 (4%)	0 (0%)	1 (2.86%)	
Hypothyroidism	No	45 (90%)	15 (100%)	33 (94.29%)	0.385
	Yes	5 (10%)	0 (0%)	2 (5.71%)	
HPL	No	48 (96%)	10 (73.30%)	29 (82.86%)	0.031
	Yes	2 (4%)	4 (26.70%)	6 (17.14%)	
BPH	No	46 (92%)	14 (93.33%)	34 (97.14%)	0.613
	Yes	4 (8%)	1 (6.67%)	1 (2.86%)	
CVI	No	48 (96%)	14 (93.33%)	34 (97.14%)	0.820
	Yes	2 (4%)	1 (6.67%)	1 (2.86%)	
COPD	No	46 (92%)	14 (93.33%)	35 (100%)	0.237
	Yes	4 (8%)	1 (6.67%)	0 (0%)	
GDS Score		15.08±5.26	14.27±4.08	14.86±4.94	0.858
Depression Group	No depression	12 (24%)	2 (13.33%)	6 (17.14%)	0.149
	Possible depression	6 (12%)	6 (40%)	10 (28.57%)	
	Certain depression	32 (64%)	7 (46.67%)	19 (54.29%)	
Sensory		11.5 (6-17)	9 (7-14)	11 (7-15)	0.075
Autonomy		10 (6-16)	11 (9-16)	11 (7-17)	0.061
PPP Activities		11 (8-17)	11 (4-15)	11 (5-17)	0.781
Social		11.08±2.05	11.13±2.5	11.57±2.55	0.611
Death		10.52±3.07	11.13±3.16	10.46±3.1	0.759
Intimacy		12 (8-16)	13 (8-16)	12 (8-18)	0.873
Total WHO-QoL OLD		22 (6-52)	26 (12-46)	22 (3-52)	0.719
HbA1c		7.55 (5.6-14.2)	6.7 (5-9.8)	7.6 (5.8-11.7)	0.520
WBC		7.51 (4.48-12.24)	8.12 (5.19-11.63)	7.78 (4.69-13.97)	0.344
Hemoglobin		12.93±1.44	14.7±1.24	14.15±1.21	<0.001
RDW		13.75 (12-18.6)	13.7 (11.8-16.2)	13.7 (11.8-19.2)	0.954
Platelet		273.78±86.88	275.93±64.05	249.2±62.76	0.294
MPV		10.65 (8.9-13.1)	10.3 (9.3-11.7)	10.6 (9.2-13)	0.081
Neutrophil		4.48 (2.03-9.07)	4.29 (2.76-7.35)	4.6 (1.83-9.4)	0.769
Lymphocyte		2.18±0.64	2.8±0.69	2.43±0.6	0.004
Monocyte		0.51 (0.31-1.09)	0.56 (0.37-0.85)	0.59 (0.37-1.19)	0.134
Glucose		146 (30-477)	165 (52-316)	156 (75-574)	0.854
BUN		15.5 (8-46)	20 (9-26)	15 (11-28)	0.432
Creatinine		0.9 (0.5-1.8)	0.8 (0.5-0.9)	0.8 (0.5-1.3)	0.024
AST		18 (11-46)	23 (10-78)	18 (10-61)	0.211
ALT		16 (6-50)	18 (9-29)	16 (9-63)	0.399
Total Cholesterol		180.74±41.57	187.6±45.66	184.26±35.98	0.825
Triglyceride		144 (53-379)	128 (51-348)	147 (55-499)	0.905
HDL		51.6±11.62	51.93±8.64	52.11±10.65	0.977
LDL		89 (30-188)	111 (46-171)	97 (40-145)	0.481
Albumin		42.58±3.66	41.13±2.92	42.94±2.8	0.200
Uric Acid		5.25 (2.1-8.6)	5.1 (3.5-6.4)	5 (2.2-9)	0.443
Spot Urine Protein/Creatinine		0.14 (0.05-2.42)	0.12 (0.05-0.38)	0.13 (0.05-1.13)	0.122

As is similar fashion in two group analysis, laboratory parameters other than hemoglobin level, lymphocyte count, and creatinine levels between groups were found not statistically significantly different between groups (Table 2). Higher hemoglobin and lymphocyte values were observed in no side effect, and side effect observed groups compared to no-SGLT patients (p < 0.001, and p = 0.004, respectively).

There were also no significant differences observed between depression levels (GDS scores) and quality of life parameters (WHOQoL scores) between side effect, no side effect, and no SGLT groups (p = 0.858 and p = 0.719, respectively). Subsections of WHOQoL scores and other variables can be seen in Table 2.

Univariate and multivariate analyses between variable groups in terms of WHOQOL-old subsection and total scores

There was no statistical difference seen between no-SGLT, no side effects, and side effect groups in terms of sensory, autonomy, PPF activities, social skills, death, intimacy, and total WHOQoL-OLD scores (p = 0.075, p = 0.061, p = 0.781, p = 0.676, p = 0.940, p = 0.873, and p = 0.719, respectively). There were statistically significantly worse outcomes observed in patients with at least one SGLT adverse effect in terms of sensory QoL scores (11 vs. 9, p = 0.026, higher sensory scores mean worse QoL). Adverse effect counts were also found to correlate with sensory scores but not with other subsections or total WHOQoL-OLD scores (p = 0.017, Table 3). The median WHOQoL subsection scores other than death, and total QoL score were found to be significantly different between no depression, possible depression, and certain depression groups (p = 0.041, p < 0.001, p < 0.001, p < 0.001, p < 0.001, p < 0.001, respectively, and for death p = 0.137). In all of the subscore categories, having depression meant lower quality of life outcomes.

**Table 3:** Comparison of variables between no-SGLT, no side effect, and SGLT side effect groups PPF: past-present-future, p: statistical significance

Variables		Sensory	P-value	Autonomy	P-value	PPF Activities	P-value	Social Skills	P-value	Death	P-value	Intimacy	P-value	Total	P-value
SGLT Usage / Side Effect	No SGLT	11,5 (6-17)	0.075	10 (6-16)	0.061	11 (8-17)	0.781	11 (6-16)	0.676	11 (4-20)	0.940	12 (8-16)	0.873	22 (6-52)	0.719
	No side effects	9 (7-14)		11 (9-16)		11 (4-15)		11 (6-15)		10 (8-18)		13 (8-16)		26 (12-46)	
	Side effect observed	11 (7-15)		11 (7-17)		11 (5-17)		12 (7-18)		11 (4-16)		12 (8-18)		22 (3-52)	
SGLT Adverse Effects	No adverse effects	9 (7-14)	0.026	11 (9-16)	0.089	11 (4-15)	0.757	11 (6-15)	0.586	10 (8-18)	0.732	13 (8-16)	0.630	26 (12-46)	0.504
	At least one effect	11 (7-15)		11 (7-17)		11 (5-17)		12 (7-18)		11 (4-16)		12 (8-18)		22 (3-52)	
Adverse Effect Count	0	9 (7-14)	0.017	11 (9-16)	0.164	11 (4-15)	0.531	11 (6-15)	0.401	10 (8-18)	0.513	13 (8-16)	0.454	26 (12-46)	0.430
	1	12 (8-15)		11 (8-17)		11 (6-17)		11 (7-18)		10 (4-16)		12 (8-18)		22 (10-52)	
	2	10 (9-13)		11 (7-16)		10 (5-16)		12 (8-15)		12 (4-16)		12 (8-16)		24 (3-49)	
	3	7 (7-7)		8 (8-8)		8 (8-8)		8 (8-8)		15 (15-15)		9 (9-9)		11 (11-11)	
Depression Group	No depression	9,5 (6-15)	0.041	13 (8-17)	<0.001	12,5 (10-17)	<0.001	12,5 (10-18)	<0.001	9,5 (4-18)	0.137	14 (11-16)	<0.001	30,5 (21-52)	<0.001
	Possible depression	11 (8-15)		11 (9-16)		11 (7-15)		11 (8-16)		10,5 (6-16)		12 (8-18)		23 (16-46)	
	Certain depression	12 (7-17)		10 (6-15)		11 (4-17)		11 (6-16)		11 (5-20)		11 (8-16)		20 (3-42)	
Poliuria	No	11 (7-15)	0.447	11 (7-17)	0.855	11 (4-17)	0.460	11 (6-16)	0.842	10 (4-18)	0.321	12,5 (8-18)	0.226	22,5 (3-52)	0.655
	Yes	11 (7-15)		11 (8-16)		11 (8-17)		11,5 (7-18)		12 (4-15)		11 (9-16)		21,5 (10-49)	
Dehydration	No	11 (7-15)	0.870	11 (8-17)	0.027	11 (4-17)	0.037	11 (6-18)	0.119	10 (4-18)	0.141	12 (8-18)	0.056	23 (10-52)	0.008
	Yes	12 (7-14)		10 (7-11)		9 (5-13)		11 (8-12)		12 (8-16)		10 (8-14)		18 (3-23)	
Weight Loss	No	11 (7-15)	0.962	11 (7-17)	0.042	11 (4-17)	0.277	11 (6-18)	0.850	11 (4-18)	0.303	11 (8-16)	0.611	23 (3-52)	0.860
	Yes	11 (7-15)		9 (8-15)		10 (6-16)		12 (8-16)		8 (5-16)		12 (8-18)		22 (11-42)	
Hypoglycemia	No	11 (7-15)	0.940	11 (7-17)	0.132	11 (4-17)	0.163	11 (6-18)	0.235	10,5 (4-18)	0.444	11,5 (8-18)	0.255	22 (3-52)	0.180
	Yes	11 (10-12)		14 (12-16)		14 (12-16)		13,5 (12-15)		8 (4-12)		14 (12-16)		36,5 (24-49)	
Urinary Infection	No	11 (7-15)	0.717	11 (8-16)	0.743	11 (4-17)	0.523	11 (6-18)	0.164	11 (4-18)	0.357	12 (8-18)	0.850	22 (10-49)	0.436
	Yes	10,5 (9-12)		11,5 (7-17)		10 (5-17)		12,5 (11-16)		8 (4-16)		11,5 (8-16)		27,5 (3-52)	

No statistically significant difference was observed in WHOQoL-OLD scores in the presence of polyuria, weight loss, hypoglycemia, and urinary infection (Table-3). Dehydration existence was negatively correlated with lower autonomy, PPF activities, and total quality of life scores, and weight loss existence was correlated with lower autonomy scores (p = 0.027, p = 0.037, p = 0.008, and p = 0.042, respectively).

**Figure 1:**
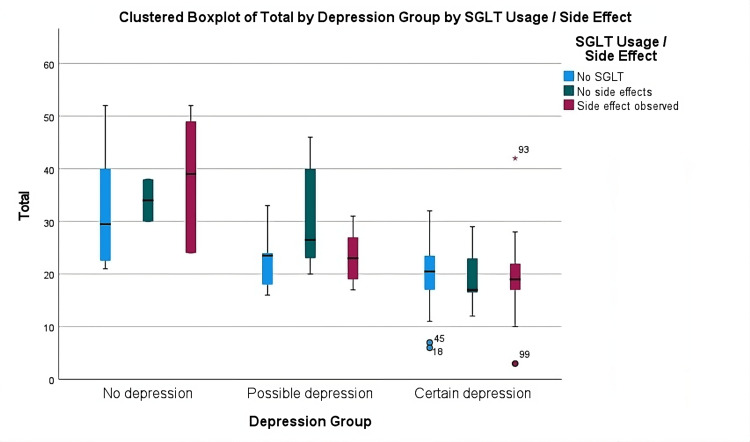
Clustered boxplot of total WHOQoL-OLD scores between depression groups by side effects o: Mild outlier, *: Extreme outlier

**Figure 2:**
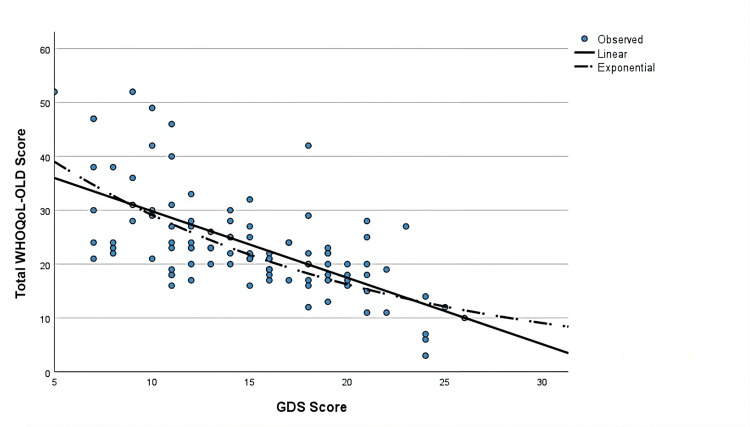
Linear and exponential models of relation between Geriatric Depression Scale scores and total quality of life scores

**Figure 3:**
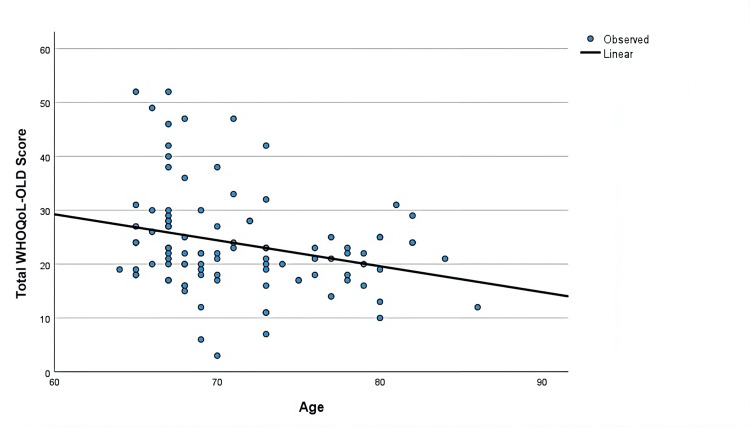
Linear model of the relation between age and total quality of life scores

A multivariate linear regression analysis with gender, age, diabetes duration, SGLT usage, side effect status, chronic disease count, GDS score, hemoglobin levels, polyuria, dehydration, weight loss, hypoglycemia, and urinary infection existence as covariates to determine the association between the variables and the Total WHOQoL-OLD Score after adjusting for confounding factors. There were no significant differences in gender, diabetes duration, chronic disease existence, chronic disease count, hemoglobin level, polyuria, weight loss, hypoglycemia, or urinary infection existence values between the groups (p = 0.280, p = 0.473, p = 0.089, p = 0.428, p = 0.525, p = 0.919, p = 0.440, p = 0.172, respectively, Table 4). While being significant in the univariate analysis, dehydration lost its significance in the multivariate analysis (p = 0.062, Table 4). There were also no differences in the total quality of life scores in SGLT usage or SGLT adverse effect existence (p=0.664). The multivariate regression model was significant (R2 = 0.519, p < 0.001), and age and depression remained as the main factors affecting the quality of life even after adjusting for potential confounders in multivariate analysis in diabetic patients (B with 95% CI = -1.233 (-1.565 - -0.902), p < 0.001 for GDS score and -0.383 (-0.675 - -0.092), p = 0.010 for age) (Table 4, Figures 1-3).

**Table 4:** Comparison of variables between no-SGLT, no side effect, and SGLT side effect groups SGLT: sodium glucose co-transporter inhibitor, GDS: geriatric depression scale

Coefficients	Unstandardized Coefficients		Standardized Coefficients			95% Confidence Interval for B	
	B	Std. Error	Beta	t	Sig.	Lower Bound	Upper Bound
Constant	71.476	12.506		5.715	<0.001	46.618	96.334
Gender	1.929	1.776	0.098	1.086	0.280	-1.6	5.458
Age	-0.383	0.147	-0.214	-2.617	0.010	-0.675	-0.092
Diabetes Duration (year)	0.073	0.101	0.059	0.72	0.473	-0.128	0.274
SGLT Usage / Side Effect	0.727	1.67	0.07	0.435	0.664	-2.592	4.046
Chronic Disease Count	1.316	0.766	0.144	1.718	0.089	-0.206	2.839
GDS Score	-1.233	0.167	-0.642	-7.399	<0.001	-1.565	-0.902
Hemoglobin	-0.453	0.568	-0.072	-0.797	0.428	-1.582	0.677
Poliuria	-1.936	3.03	-0.082	-0.639	0.525	-7.959	4.088
Dehydration	-6.042	3.195	-0.163	-1.891	0.062	-12.392	0.309
Weight Loss	-0.320	3.146	-0.011	-0.102	0.919	-6.572	5.933
Hypoglycemia	4.317	5.559	0.064	0.777	0.440	-6.732	15.367
Urinary Infection	5.736	4.161	0.119	1.379	0.172	-2.534	14.006

## Discussion

Diabetes management in older adults is a challenging process due to accompanying geriatric syndromes, comorbid diseases, increased risk of hypoglycemia, and microvascular complications. In addition, there are many problems in this patient population, such as fluctuations in blood sugar levels, decreased cognitive functions and skills, decreased self-care associated with depression, difficulty in compliance with treatment, and sarcopenia [14,15]. In this patient population, the goals should be to maintain quality of life and minimize hypoglycemia and drug side effects [16,17]. In this study, no significant difference was found between the groups in terms of gender, duration of diabetes, presence of chronic disease, number of chronic diseases, and GDS scores. There were also no significant differences observed between depression levels (GDS scores) and quality of life parameters (WHOQoL scores) between side effects, no side effects, and no SGLT groups.

In this cross-sectional study, patients using SGLT were found at a younger age than those who did not. This may be related to the fact that physicians are more reluctant when starting medication for older adults. There are few studies to date on the use of SGLT-2 inhibitors in the geriatric population. To this date, there are few studies on the use of SGLT-2 inhibitors in older adults [18]. SGLT-2 inhibitors offer the advantage of the ease of oral administration, multiple positive effects on blood glucose, HT, and weight loss, as well as cardioprotective and renoprotective effects. However, due to insufficient data on its long-term use, clinicians are hesitant to use it in geriatric patients [19]. SGLT-2 inhibitors have also been reported as a safe and effective treatment option in the geriatric population, but closer monitoring and cautious use are recommended, especially in more frail subjects and older adults, in terms of the risk of complications compared to younger older adults [20,21].

Some adverse outcomes in older patients with diabetes are of particular concern to clinicians. Hypoglycemia, in particular, is a feared condition in older diabetic patients [22]. The risk of hypoglycemia was found to be similar for empagliflozin and dapagliflozin compared to placebo [23]. The possible effects of SGLT-2 inhibitors, such as weight loss, might be beneficial in the younger population, especially in patients with heart failure and obesity, and improve quality of life. However, cachexia, sarcopenia, and hypovolemia are important problems in geriatric patients, and they may be more sensitive to the effects of SGLT-2 inhibitors [24].

Studies have shown that SGLT-2 inhibitors reduce adipose tissue mass while maintaining lean body mass [24]. Muscle loss associated with excessive weight loss is particularly undesirable in older adults; the EMPA-ELDERLY study is ongoing to examine the change in the effects of SGLT-2 inhibitors on muscle mass in the geriatric population [25]. In patients treated with SGLT-2 inhibitors, dehydration, and osmotic diuresis may occur due to volume depletion, and although geriatric patients are more at risk in this respect, studies have not found significant differences compared to placebo [26]. There was no significant difference in polyuria, weight loss, hypoglycemia, or the presence of urinary infection in our study.

Depression has a significant impact on the quality of life of individuals across various populations. Several studies have examined the relationship between depression and quality of life in different contexts, including diabetes and hemodialysis. These studies consistently demonstrate that depression is associated with lower quality of life. In a study on individuals with diabetes, the prevalence of depression was found to be higher in those with diabetes compared to those without diabetes and found that depression in individuals with diabetes was associated with lower quality of life [27]. Similarly, in a study on older adults on hemodialysis, depression was found to be highly prevalent and was associated with lower quality of life [28]. These studies consistently demonstrate that depression is associated with lower quality of life; this was also true in our study, according to multivariate analysis results.

In this study, no statistically significant difference was observed in WHOQoL-OLD scores in the presence of polyuria, weight loss, hypoglycemia, and urinary infection. Dehydration existence was negatively correlated with lower autonomy, PPF activities, and total quality of life scores, and weight loss existence was correlated with lower autonomy scores in the univariate analysis. There were no significant differences in no differences in the total quality of life scores in the SGLT usage group or SGLT adverse effect group in the multivariate analysis. While significant in the univariate analysis, dehydration lost its significance in the multivariate analysis. Age and depression remained the main factors affecting the quality of life even after adjusting for potential confounders in multivariate analysis in diabetic patients. We did not find worse QoL scores in the group with side effects of SGLT-2 inhibitors.

## Conclusions

In conclusion, in our study, the possible side effects of SGLT-2 inhibitors did not impair the quality of life in older adult patients who are in a more vulnerable risk group in terms of adverse effects. We believe that this may be related to the advantage of oral use, blood pressure regulation in addition to blood glucose regulation, and primarily cardioprotective and renoprotective effects. Studies on the use of SGLT-2 inhibitors in the geriatric population are limited in the literature, and we suggest that our results should be supported by new studies with more participants in this field.
